# Complex Pediatric Foot Injuries Secondary to Entrapment in Motorcycle Spokes: A Series of 56 Cases

**DOI:** 10.7759/cureus.111099

**Published:** 2026-06-18

**Authors:** Alberto Daniel Navarro Vergara, Romina Blasco Segales, Claudia Rios Mareco, Angel Cristaldo Castillo

**Affiliations:** 1 Orthopedics and Traumatology, Hospital de Trauma “Manuel Giagni”, Asuncion, PRY; 2 Orthopedics and Traumatology, Hospital de Trauma "Manuel Giagni", Asuncion, PRY

**Keywords:** child, child safety, foot injuries, motorcycle injuries, pediatric trauma, wheel-spoke injuries

## Abstract

Objectives: To describe the clinical presentation, severity, anatomical involvement, and initial management of pediatric foot and ankle injuries secondary to motorcycle wheel-spoke entrapment.

Methods: A retrospective, observational, descriptive study was conducted on a consecutive series of pediatric patients with foot and ankle injuries caused by motorcycle wheel-spoke entrapment, treated at a referral trauma center between January 2023 and December 2025. Demographic characteristics, anatomical distribution according to the Zwipp zonal classification, injury severity based on the Zwipp score and Muzzammil classification, affected structures, treatment performed, number of surgical procedures, and length of hospital stay were analyzed.

Results: Fifty-six patients were included. High-severity injuries (Zwipp score ≥5) were identified in 22 patients (39.3%), while type IIIB and IIIT injuries accounted for 84% of cases according to the Muzzammil classification. Bone exposure was documented in 30 patients (53.6%), and open fractures in 21 (37.5%). The tarsometatarsal region was the most frequently affected anatomical site. Management required a median of three surgical procedures (interquartile range (IQR), 2-3), and soft-tissue coverage using skin grafts and/or flaps was required in 18 patients (32.1%).

Conclusions: Motorcycle wheel-spoke entrapment is associated with severe foot and ankle injuries in children, characterized by extensive soft-tissue damage, osseous and tendinous involvement, and the frequent need for multiple surgical procedures. This series provides a detailed characterization of the anatomical patterns, severity, and initial management of this uncommon but potentially devastating mechanism of pediatric trauma.

## Introduction

Foot and ankle trauma caused by entrapment in motorcycle wheel spokes represents a distinctive mechanism of injury in the pediatric population, capable of producing severe damage to both osseous and soft-tissue structures [1,2]. Owing to the smaller size of the pediatric foot and ankle, entrapment within the rotating wheel mechanism may generate combined crushing, traction, and torsional forces, resulting in a wide spectrum of lesions ranging from superficial wounds to complex injuries involving multiple anatomical structures [2]. In the most severe cases, multidisciplinary management may be required to preserve limb viability and address complex soft-tissue and osseous injuries [3].

Motorcycle-related injuries have become an increasing public health concern in developing countries, where motorcycles are widely used and compliance with traffic safety regulations remains limited [3]. In Paraguay and other countries with similar socioeconomic characteristics, the transport of children on motorcycles remains a common practice [4,5]. Consequently, motorcycle wheel-spoke injuries continue to represent a preventable source of severe foot and ankle trauma in the pediatric population.

This study presents a consecutive series of pediatric patients with foot and ankle injuries secondary to motorcycle wheel-spoke entrapment treated at a referral pediatric trauma center in Asunción, Paraguay. The aim of this study was to describe the clinical presentation, injury severity, anatomical involvement, and initial management of pediatric motorcycle wheel-spoke injuries treated at a referral trauma center.

## Materials and methods

Study design

A retrospective, observational, descriptive study was conducted, consisting of a consecutive series of pediatric patients with foot and ankle injuries secondary to motorcycle wheel-spoke entrapment, treated at a pediatric trauma referral center between January 2023 and December 2025.

The present study was derived from a larger institutional database designed to investigate pediatric foot and ankle injuries secondary to motorcycle wheel-spoke entrapment. The current analysis focused on the clinical presentation, severity, anatomical involvement, and initial management of these injuries.

Study participants

Patients aged 16 years or younger who had not received definitive surgical treatment at another institution were eligible for inclusion. Cases involving pathological bone conditions or pre-existing morphological abnormalities of the foot and ankle were excluded to avoid potential interference with injury characterization and severity assessment.

Data collection and variables

The variables analyzed included demographic characteristics (age and sex), injury laterality, and anatomical involvement. The affected anatomical regions were recorded according to the Zwipp zonal classification [6], which divides the foot and ankle into five anatomical regions: the tibiotalar joint, talus, calcaneus, transverse tarsal joint, and tarsometatarsal joints. This classification was used to characterize the anatomical distribution and extent of injuries.

Injury severity was assessed using the Zwipp score [6] and the Muzzammil classification [7]. The latter is a classification system specifically developed for pediatric wheel-spoke injuries and was used to characterize the severity of soft-tissue and associated structural involvement.

The affected anatomical structures were documented at the initial evaluation, including osseous, tendinous, soft-tissue, and neurovascular injuries. The number of surgical procedures performed until hospital discharge, the definitive orthopedic treatment, and the requirement for soft-tissue coverage by the plastic surgery service using skin grafts and/or flaps were also recorded.

In addition, the presence of immediate vascular complications and the length of hospital stay were documented. Hospital stay was calculated from the day of admission to the institution until discharge.

Statistical analysis

A descriptive statistical analysis was performed. Quantitative variables were expressed according to data distribution, using mean ± SD or median with interquartile range, as appropriate. Categorical variables were summarized as absolute frequencies and percentages.

Ethical considerations

The study was conducted in accordance with ethical standards and received approval from the Institutional Ethics Committee (ME 2710/501). Data were anonymized and managed confidentially, following the ethical principles outlined in the Declaration of Helsinki.

## Results

**Figure 1:**
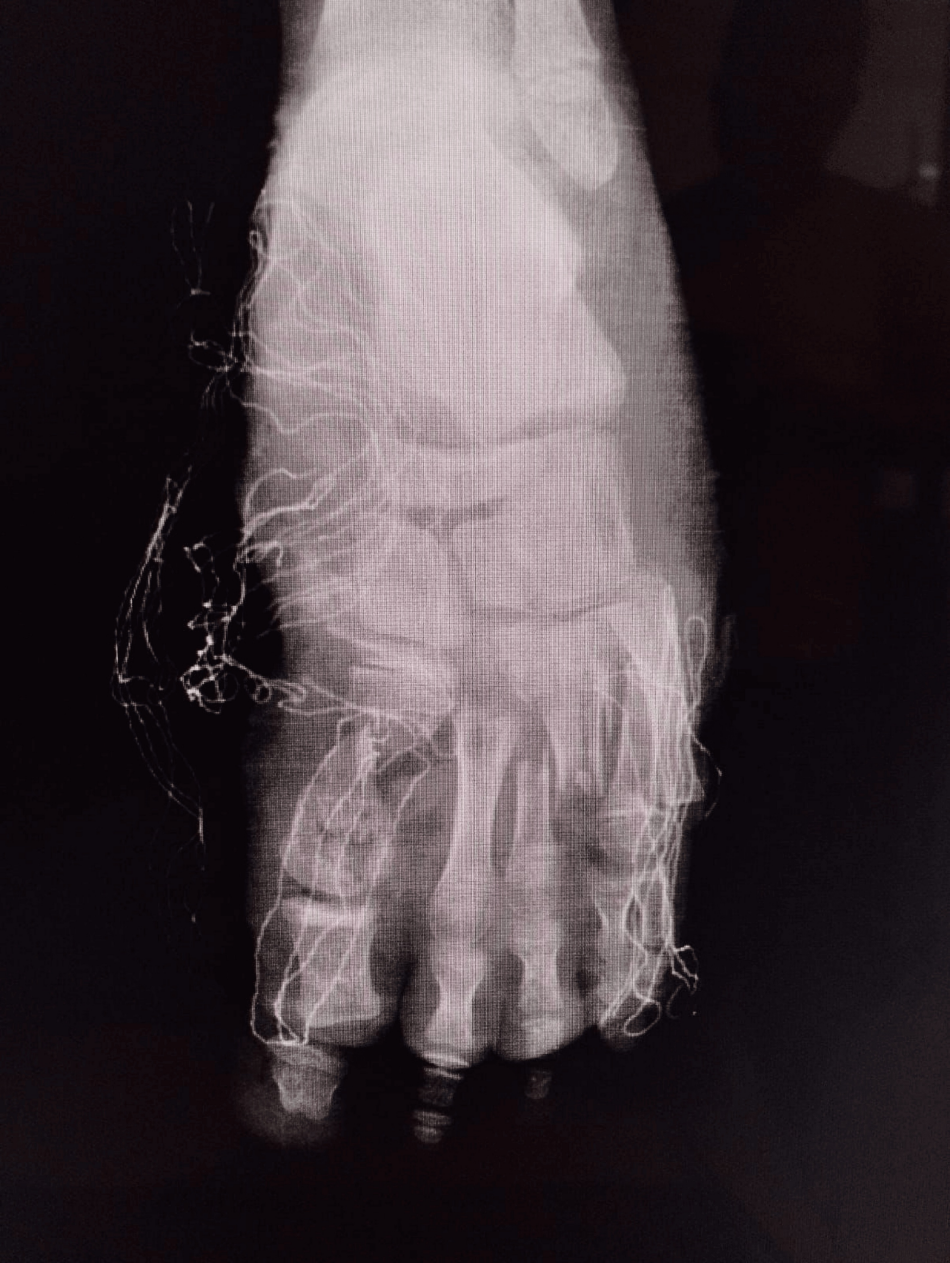
Anteroposterior radiograph showing tarsometatarsal involvement (Zwipp zone 5). Anteroposterior radiograph of the foot demonstrating involvement of the tarsometatarsal region (Zwipp zone 5), illustrating a complex pediatric foot injury resulting from motorcycle wheel-spoke entrapment.

Fifty-six pediatric patients with foot and ankle injuries secondary to motorcycle wheel-spoke entrapment were included in the study (Figure 1). The mean age was 8.8±4.1 years. Male patients predominated, accounting for 39 cases (69.6%) of the cohort. The demographic characteristics of the study population are summarized in Table 1.

**Table 1:** Demographic characteristics of the study population (n=56). Data are presented as mean ± SD, median (interquartile range), or number (%), as appropriate.

Variable	Result
Age (years), mean ± SD	8.8±4.1
Age (years), median (IQR)	9 (6-13)
Male sex, n (%)	39 (69.6)
Female sex, n (%)	17 (30.4)

With regard to laterality, the left side was affected slightly more frequently than the right, accounting for 30 (53.6%) and 26 (46.4%) cases, respectively. No cases of bilateral involvement were identified.

According to the Zwipp zonal classification, the tarsometatarsal region (zone 5) was the most frequently affected anatomical area, being involved in 25 patients (44.6%), followed by the talus (zone 2) in eight cases (14.3%) and the calcaneus (zone 3) in seven cases (12.5%). Injuries involving more than one anatomical region were identified in 16 patients (28.6%).

According to the Zwipp score, 22 patients (39.3%) presented with high-severity injuries (score ≥5). The Muzzammil classification showed a predominance of type IIIB injuries, identified in 32 patients (57.1%), followed by type IIIT injuries in 15 cases (26.8%) and type IV injuries in six patients (10.7%). Less severe injuries (type II) were observed in only three patients (5.4%).

Bone exposure was documented in 30 patients (53.6%). Open fractures were identified in 21 patients (37.5%). Detailed characteristics of injury laterality, anatomical distribution, and severity are summarized in Table 2.

**Table 2:** Characteristics of foot and ankle injuries according to laterality, anatomical distribution, and severity (n=56). *Combined zones correspond to injuries that affected more than one anatomical region of the foot and/or ankle.

Variable	n (%)
Laterality	
Right	26 (46.4)
Left	30 (53.6)
Anatomical region according to Zwipp zoning	
Tarsometatarsal joints (zone 5)	25 (44.6)
Talus (zone 2)	8 (14.3)
Calcaneus (zone 3)	7 (12.5)
Combined zones*	16 (28.6)
Muzzammil classification	
Type II	3 (5.4)
Type IIIB	32 (57.1)
Type IIIT	15 (26.8)
Type IV	6 (10.7)
Bone exposure	
Present	30 (53.6)
Absent	26 (46.4)

**Figure 2:**
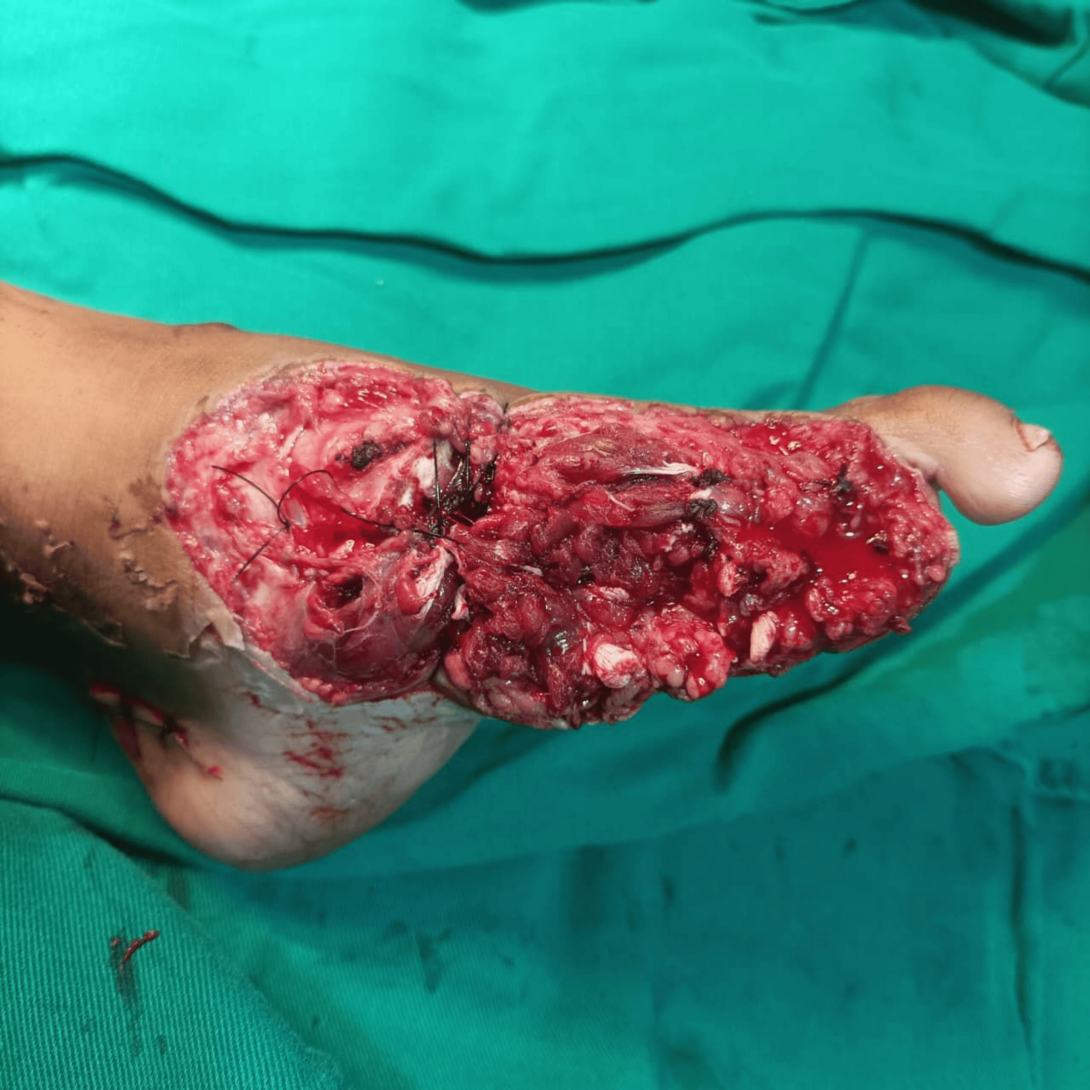
Severe foot and ankle injury caused by motorcycle wheel-spoke entrapment. Clinical photograph demonstrating a severe foot and ankle injury resulting from motorcycle wheel-spoke entrapment in a pediatric patient. The image illustrates the extensive soft-tissue damage associated with this mechanism of trauma.

Among the osseous injuries identified, the metatarsals and calcaneus were the most frequently affected structures (Figure 2), with fractures involving 15 patients (26.8%) at each location. Phalangeal fractures were observed in 11 patients (19.6%). Less frequently, injuries involved the distal tibia (three patients), the talus (two patients), and the navicular (one patient). Multiple osseous injuries within the same patient were identified in several cases.

Achilles tendon injuries were documented in 18 patients (32.1%), representing the most common tendinous lesion. In several patients, tendon injuries occurred in association with adjacent osseous involvement. Amputations were required in six patients (10.7%), reflecting the devastating nature of the most severe injuries observed in this cohort.

Management of these injuries frequently required multiple surgical procedures. A median of three operations (interquartile range (IQR), 2-3) was performed until hospital discharge. Soft-tissue coverage by the plastic surgery service was required in 18 patients (32.1%), using skin grafts and/or local or regional flaps as part of multidisciplinary management.

Definitive orthopedic treatment was tailored according to injury severity and the anatomical structures involved. Treatment strategies included wound debridement and closure, fracture stabilization, immobilization, and osseous debridement and curettage when required. The distribution of definitive treatments is summarized in Table 3.

**Table 3:** Definitive orthopedic treatment performed in patients with foot and ankle injuries (n=56). *Combined treatments involved two or more orthopedic procedures (e.g., pins combined with suturing or bone remodeling).

Definitive treatment	n (%)
Soft tissue repair	16 (28.6)
Kirschner fixation	16 (28.6)
Immobilization alone	14 (25.0)
Osseous debridement and curettage	6 (10.7)
Combined orthopedic procedures*	4 (7.1)

No immediate vascular complications related to the injury or initial surgical treatment were documented. The median length of hospital stay was six days (IQR, 3-12).

## Discussion

This study describes a consecutive series of pediatric foot and ankle injuries secondary to motorcycle wheel-spoke entrapment, a mechanism of trauma that remains frequent in countries where motorcycles are widely used for transportation. Although the foot and ankle are not among the most commonly injured anatomical regions in pediatric trauma [3], wheel-spoke entrapment may result in disproportionately severe injuries, characterized by extensive soft-tissue damage, osseous involvement, and the frequent need for surgical management [6,7].

One of the most relevant findings of this series is the predominance of severe injuries, as demonstrated by both the Zwipp score and the Muzzammil classification, with a high proportion of type IIIB and IIIT lesions. This pattern reflects the high-energy nature of the injury mechanism, in which the child's foot becomes entrapped within a rotating wheel system, generating combined torsional, crushing, and avulsion forces [8]. The high frequency of bone exposure and open fractures observed in this cohort further supports the severity of these injuries and helps explain the complexity of their subsequent management. In addition, amputations were required in a subset of patients, emphasizing the potentially devastating consequences of this mechanism of trauma.

From an anatomical perspective, the tarsometatarsal region was the most frequently affected site, followed by injuries involving other regions of the hindfoot and multizonal lesions. This pattern may be related to the mechanism of wheel-spoke entrapment, in which the relatively small size of the pediatric foot facilitates its introduction into the rotating wheel system [4,8]. The involvement of multiple anatomical regions observed in nearly one-third of patients highlights the potential extent of these injuries and underscores the importance of thorough initial assessment and careful surgical planning [9].

Previous reports from Asia and Africa have described injury patterns similar to those observed in the present series, characterized by severe foot and ankle trauma, frequent soft-tissue compromise, and the need for repeated surgical procedures [1,10]. These findings suggest that motorcycle wheel-spoke entrapment produces a relatively consistent pattern of injury across different geographic regions where motorcycles are widely used for transportation. In contrast, reports from high-income countries are less frequent, reflecting the lower incidence of this mechanism of injury in those settings [8]. Within Latin America, available evidence remains scarce, highlighting the importance of reporting regional experiences to improve understanding of the epidemiology and clinical characteristics of these injuries in the pediatric population [3-5].

Injuries caused by wheel-spoke entrapment have traditionally been described in association with bicycles, particularly in the pediatric population [11]. Classic reports, including those of Moraes and Abreu, generally describe lower-energy injuries predominantly affecting the soft tissues, with relatively infrequent osseous involvement and a low incidence of major reconstructive procedures [11]. In contrast, the present series demonstrated a high frequency of bone exposure, open fractures, tendon injuries, and amputations, highlighting the greater severity that may be associated with motorcycle wheel-spoke trauma.

More recent comparative studies, such as that by Mak et al. [12], which evaluated bicycle- and motorcycle-related wheel-spoke injuries in children, have demonstrated that motorcycle-associated trauma is substantially more severe. Reported findings include deeper soft-tissue injuries, frequent Achilles tendon involvement, a greater need for surgical intervention, and longer hospital stays. These observations are consistent with the findings of the present series, in which tendon injuries, bone exposure, open fractures, and the requirement for multiple surgical procedures were common. Collectively, these data support the concept that, although the mechanism of entrapment is similar, motorcycle wheel-spoke injuries are associated with considerably greater tissue damage than the classic bicycle spoke injuries described in the pediatric literature.

Management of these injuries frequently required multiple surgical procedures, as reflected by the median of three operations performed before hospital discharge. The need for repeated interventions should not be interpreted as a failure of initial treatment, but rather as a characteristic feature of severe foot and ankle injuries. In these cases, sequential debridement, soft-tissue management, skeletal stabilization, and, when necessary, reconstructive procedures are often required to preserve limb viability and minimize the risk of complications [8,9].

The frequent involvement of the plastic surgery service, required in approximately one-third of patients, underscores the substantial soft-tissue compromise associated with this injury mechanism. Soft-tissue coverage using skin grafts and/or flaps allowed closure of complex defects and protection of exposed anatomical structures, forming an essential component of multidisciplinary management. These findings highlight the importance of treating such injuries in referral centers with access to reconstructive techniques and experience in complex pediatric trauma [13].

The variety of orthopedic treatment strategies employed in this series reflects the heterogeneity of injury patterns encountered following motorcycle wheel-spoke entrapment [14]. Given the wide spectrum of osseous, tendinous, and soft-tissue injuries observed, therapeutic decisions must be individualized according to injury severity and the anatomical structures involved. In pediatric patients, treatment planning should prioritize limb preservation while taking into account the unique characteristics and growth potential of the immature foot [15].

The findings of the present study should be interpreted in light of its limitations. The analysis focused on the acute presentation and initial management of these injuries, without evaluating long-term functional outcomes. Complex foot and ankle injuries in the pediatric population may be associated with late sequelae, including joint stiffness, gait abnormalities, residual pain, and growth-related deformities, which may not become evident until several months or years after the initial trauma [13,14].

Study limitations and clinical implications

This study has several limitations that should be considered when interpreting its findings. First, its retrospective design is subject to the limitations inherent to this type of investigation, including reliance on the completeness and accuracy of medical records. In addition, follow-up was limited to the acute phase of treatment, precluding the systematic assessment of medium- and long-term functional outcomes and the identification of late sequelae related to skeletal growth.

Another relevant limitation is the absence of standardized functional outcome measures. Although this precludes an objective evaluation of long-term function, the primary aim of the present study was to characterize the clinical presentation, severity, anatomical involvement, and initial management of pediatric motorcycle wheel-spoke injuries. Functional assessment was therefore beyond the scope of the current analysis and should be addressed in future investigations.

Despite these limitations, this series provides clinically relevant information regarding a severe and relatively underreported mechanism of injury in the pediatric population. The findings reinforce the importance of managing these injuries in referral centers with multidisciplinary teams experienced in complex pediatric trauma and reconstructive procedures.

From a clinical perspective, the present study highlights the importance of meticulous initial evaluation, comprehensive assessment of soft-tissue damage, and individualized surgical planning. Given the frequency of severe soft-tissue compromise, open fractures, tendon injuries, and the need for multiple surgical procedures, early multidisciplinary management appears essential to optimize acute care. Furthermore, these findings emphasize the importance of preventive strategies aimed at reducing unsafe motorcycle-related transportation practices involving children.

## Conclusions

In this series, motorcycle wheel-spoke entrapment was associated with severe foot and ankle injuries in children, frequently involving extensive soft-tissue compromise as well as osseous and tendinous structures. These injuries often required multiple surgical procedures, including soft-tissue coverage procedures. This study describes the anatomical patterns, severity, and initial management of this potentially devastating mechanism of pediatric trauma.
